# Multicentric Spinal Pilocytic Astrocytoma Presenting with Syringomyelia

**DOI:** 10.7759/cureus.13353

**Published:** 2021-02-15

**Authors:** Ahad A Alanazi, Young-shin Ra

**Affiliations:** 1 General Medicine, King Saud University, Riyadh, SAU; 2 Paediatric Neurosurgery, Asan Medical Center, Seoul, KOR

**Keywords:** neuro oncology, pilocytic astrocytoma, spinal cord tumors, syringomyelia, neuro spine, neuro-surgery

## Abstract

Pilocytic spinal cord astrocytomas make up 21% of intramedullary tumors. Only 20% of those tumors are associated with syringomyelia. To our knowledge, this is the first report of an adult presenting with multiple spinal pilocytic astrocytomas associated with syringomyelia. We report a case of a 27-year-old woman who had neck and arm pain for months. She underwent cervical magnetic resonance imaging (MRI) that demonstrated a syrinx from C2 and extending to C6. A coronal view of the MRI showed multiple mural nodules. Total excision of multicentric nodules within a cyst was performed with an uneventful intraoperative and postoperative period and the patient was discharged home with moderate right-hand numbness.

## Introduction

Spinal cord gliomas are rare lesions, comprising only 8%-10% of all primary spinal cord tumors. In adults, the most common histopathologic type of spinal cord tumors is ependymoma (60%-70%), followed by astrocytoma (30%-40%) [1-4]; 85%-90% of astrocytomas are low grade (World Health Organization (WHO) grade I) and 20% of them are associated with syrinx formation. Multiple spinal pilocytic astrocytomas are considerably rare lesions [5], and when coexisting with the presence of a syrinx, then become a unique case for reporting, along with possibly gaining a better understanding of the diagnosis and management of primary intramedullary tumors.

## Case presentation

**Figure 1:**
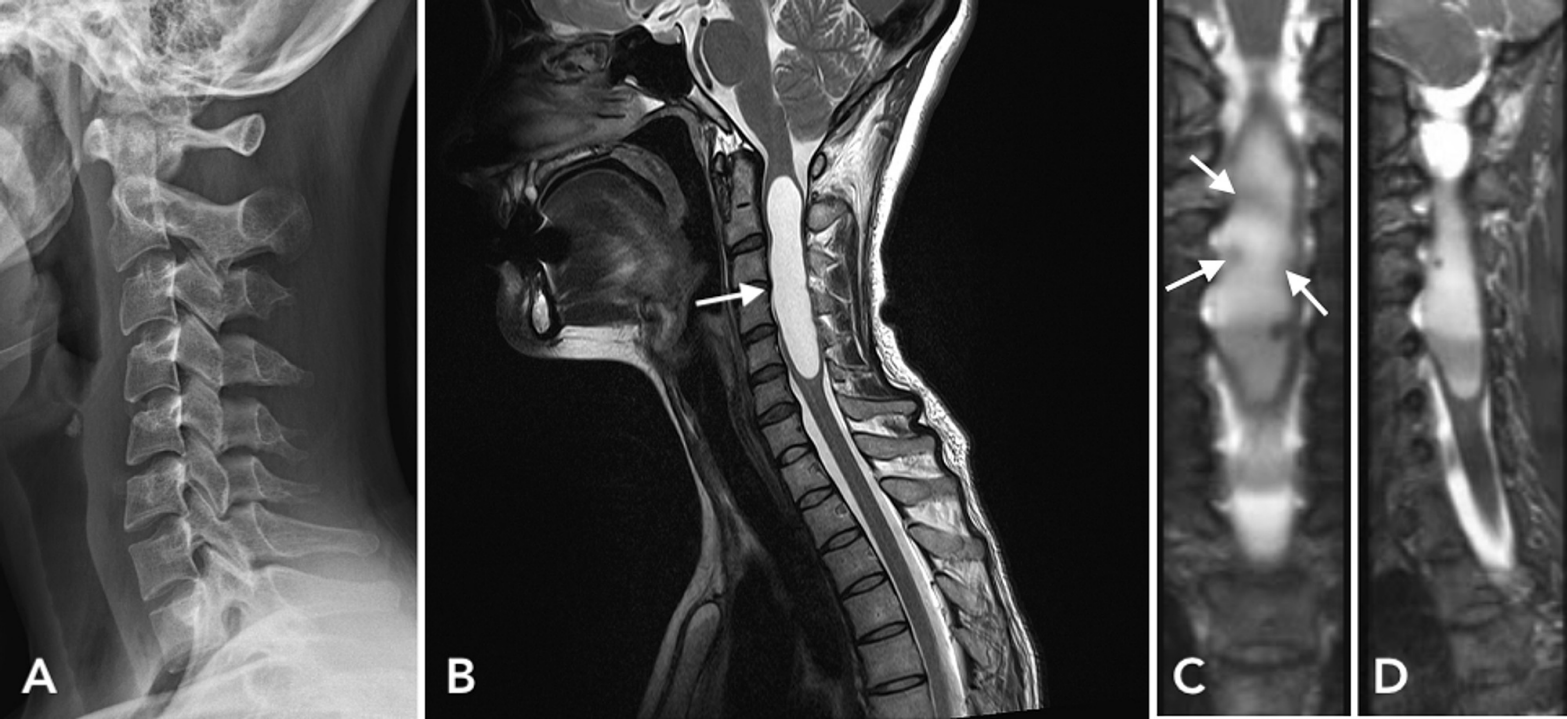
Cervical X-ray showing widening of the spinal canal between C3-C5 (A). Sagittal spinal MRI showing large syrinx that extends from C2 to C6 (B). T2 weighted image (T2WI)-MRI coronal view shows multiple mural nodules with some contrast enhancement (arrows) (C), sagittal view (D)

**Figure 2:**
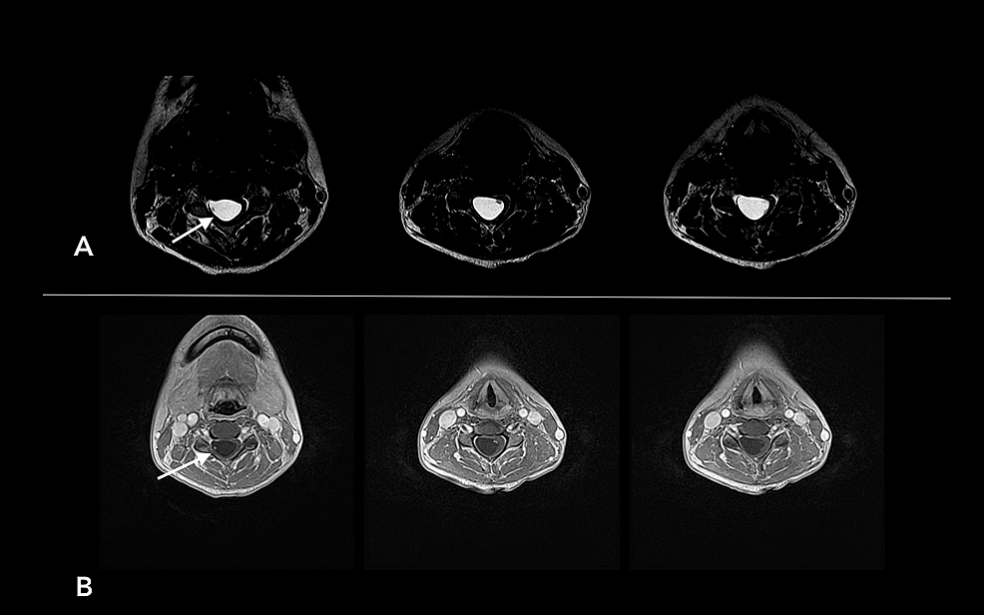
Axial MRI shows spinal intramedullary mass that appears hyperintense on T2 (arrow) (A) and hypointense on T1 (B)

A 27-year-old woman presented with posterior neck and right arm pain for several months. On examination, she showed intact motor and sensory functions. The deep tendon reflexes of her right biceps were slightly hyperactive. Cervical X-rays showed a widening of the cervical spinal canal between C3 and C5 vertebrae as well as mild degenerative spondylosis in the C6-C7 intervertebral space with kyphotic curvature (Figure 1A). Cervical spine MRI shows a large syrinx from C2 to C6 vertebral levels without cerebellar tonsillar herniation (Figure 1B). Coronal view of T2 weighted images (T2WI) shows multiple minuscule, mural nodules with some contrast enhancement (Figure 1C). Axial MRI shows a spinal intramedullary mass that appears hyperintense on T2 and hypointense on T1 (Figure 2).

**Figure 3:**
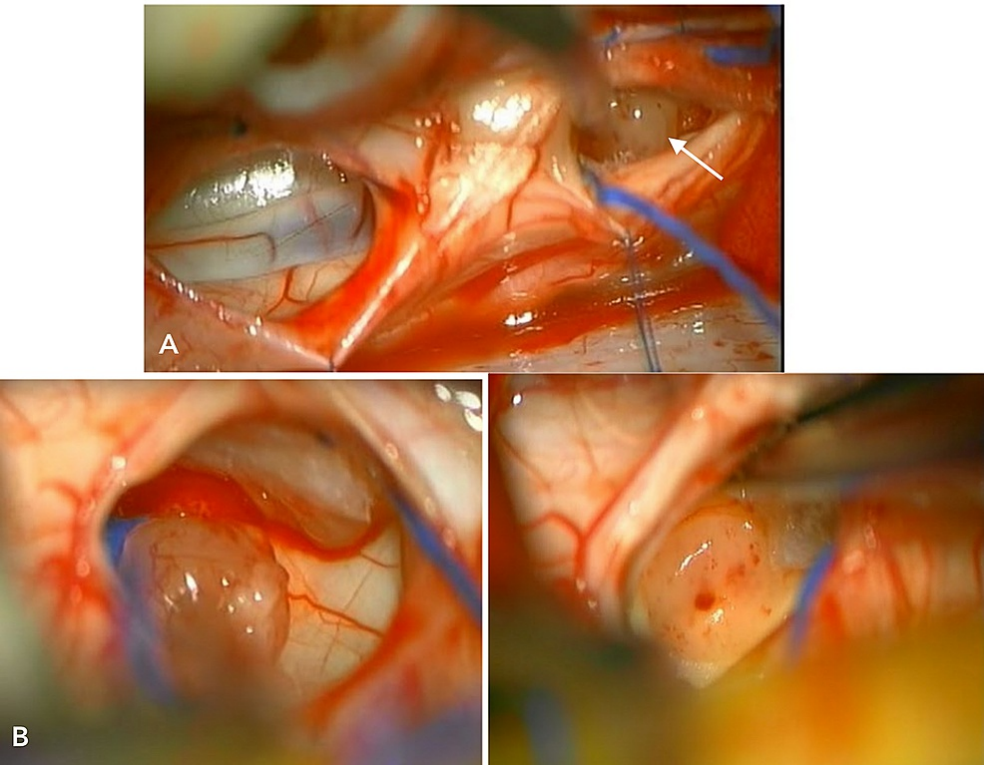
Intra-operative images: dura incision at C3 and C4 levels. The right side of the spinal cord at C3 level showing tumor nodule (arrow) (A), two nodules at C4 showing the same tumor characteristic of the mural nodule at the C3 spinal level (B)

**Figure 4:**
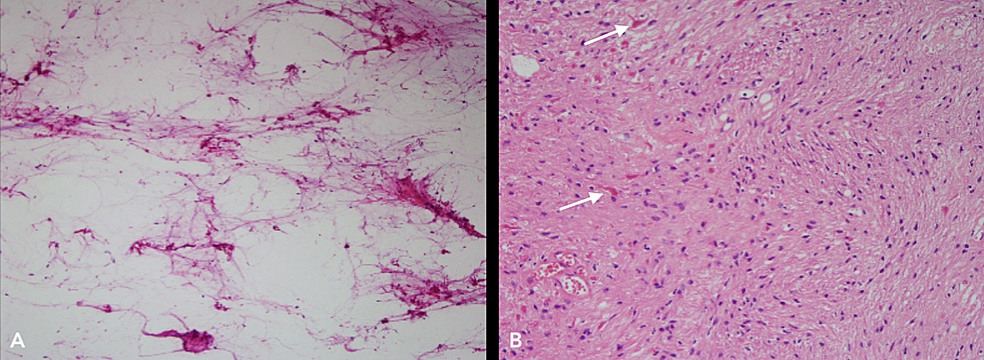
Histopathology images biphasic, with alternating compact and loose/microcystic growth pattern (A), High power view of pilocytic astrocytoma with Rosenthal fibers (arrows) (B)

She was brought to the operating room under the impression of a cyst with mural tumorous lesions, such as hemangioblastomas. During the operation, her head was fixed in place in a prone position. Motor and sensory evoked potentials were monitored continuously during the operation. With a midline skin incision from C2-C5 spine level, right paravertebral muscles were dissected from the spinous process of the vertebrae and then a hemilaminectomy was done at C3 and C4 vertebrae. The dura was slightly tense but intact in color and shape. As dura was incised and reflected in the paramedian line, an enlarged spinal cord came into view. It was bulged out and there was no abnormal vessel in size and color. With Arachnoid dissection, a pial surface of the dorsal roots exit zone, the right side of the spinal cord at C3 level was incised 7 mm-long under the microscope and a cyst was dissected through the Lissauer’s tract gently. Then yellowish colored fluid gushed out through a window of the cyst. Inside of the cyst, a dark gray-yellowish, 3 mm-diameter, solid and nodular tissue came into view without abnormal vessels which are common in hemangioblastomas. The wall of the cyst cavity was clear without tumor invasion. The tumor nodule was soft and well-demarcated with the surrounding spinal cord parenchyma. It was removed with microscissors carefully. Then another pial incision was made at the right side of the spinal cord at the C4 lower spinal level 1 cm below the previous one. The cyst was dissected again and two nodules were found at the lateral side of the cyst. They showed the same tumor characteristic of the mural nodule at the C3 spinal level (Figure 3). Complete resection of three singular pilocytic astrocytomas (PAs) was performed successfully and without events in intraoperative monitoring. Dura was repaired in a watertight fashion and the wound was closed layer by layer. Postoperative recovery was uneventful and she was discharged home with some right-hand numbness on the 6th postoperative day. Pathology nodular tissue was reported as pilocytic astrocytoma (Figure 4).

**Figure 5:**
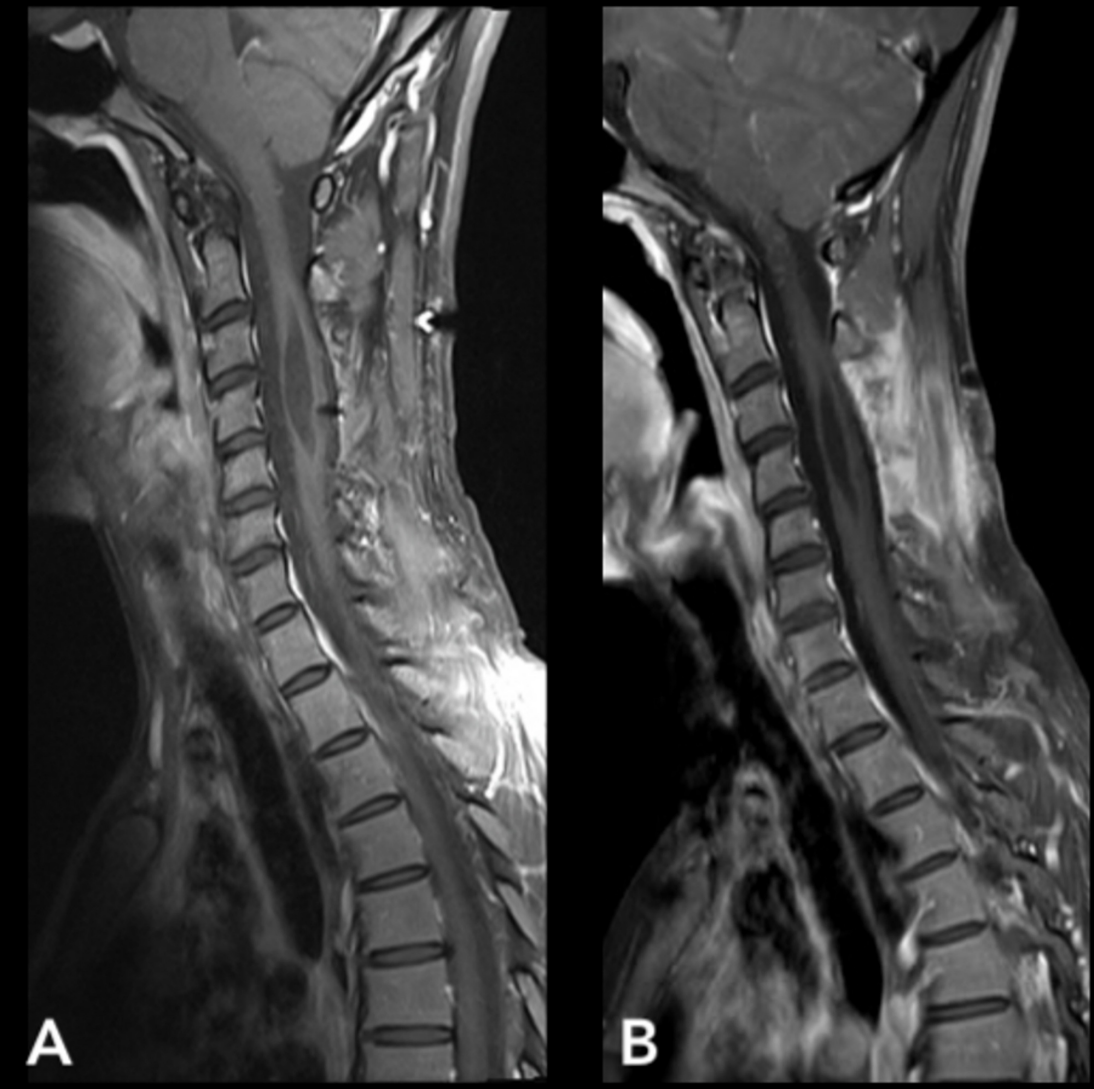
Post-operative imaging: T1 weighted images (T1WI) immediately after surgery (A), and after one year (B)

Considerations for adjuvant therapy got deferred until any tumor recurrence on following imaging study and after the discussion at the multidisciplinary pediatric neuro-oncology board. There was no tumor recurrence on the postoperative MRI images immediately after surgery and at a one-year interval (Figure 5). 

## Discussion

Intramedullary spinal cord tumors (IMSCT) are rare entities that comprise 6%-8% of all central nervous system tumors and 20%-25% of spinal cord tumors [1,2]. Ependymomas account for the majority of cases - 60%-70% in the adult population, whereas astrocytomas are the second most common accounting for 30%-40% and they are more common in children than in adults [5-7]. 

Due to their infrequent incidence as well as their unknown etiology and proximity to the spinal cord, IMSCTs are considered a challenge to neurosurgeons in both their diagnosis and their management. They carry a great risk of resulting in neurological deficits; prompt optimal treatment, including the goal of total tumor resection, is advised for better management outcome [8,9]. To our knowledge, there are no cases reported in the literature with multiple spinal pilocytic astrocytomas in the presence of a syrinx. 

PA are benign tumors according to the WHO 2016 classification of gliomas; their incidence in the adult population is unknown, but they are considered one of the most common intramedullary spinal cord tumors in pediatrics [7,10-12]. Spinal PAs are unique among astrocytomas for tending to displace the spinal cord rather than infiltrating it, interestingly, those types of tumors are more likely to be associated with syrinx than infiltrative ones and have a better prognosis [6,7]. Syrinx is the general term that describes a cystic cavity in the spinal cord, syringomyelia is a syrinx that is eccentric to the central spinal canal, those two terms are difficult to distinguish from one another [13-15]. The cervical region is the second most common location, after thoracic, where spinal PAs are found, and rarely do they occur in the lumbar region. The cervical location of IMSCT achieved more complete resection than any other location of IMSCTs in the literature, and the surgical approach for these cases is usually laminotomy with a reconstruction of the posterior spinal column [9]. The two most significant factors determining the outcome of IMSCT are preoperative neurological status and tumor histology [6,7,13]. Tumor histology affects the outcome because it can predict both the resectability and the recurrence of the tumor [6]. Even though the clinical features of IMSCT are dependent on the location, tumor extent, and growth rate, the most common presenting symptom in spinal cord astrocytoma is pain, and in the case of spinal PAs specifically, they tend to grow insidiously with no apparent neurologic deficits [2,7,8]. The gold standard treatment remains to be complete surgical resection only. Resection of IMSCTs requires a careful examination of the tumor-spinal cord interface and aiming for a complete resection without compromising the spinal neural tract [11,13]. Complete resection is advised for tumors with a clear plane of dissection (POD). It is more likely that a neurological deficit would occur if that plane is not identified and the surgeon proceeded with a total resection [16]. The role of radiotherapy in low-grade astrocytoma is controversial and only advised in high-grade lesions, and they are generally not recommended in PA [2,6,9,17]. Finally, having an identifiable POD and the histopathological diagnosis of PA makes this case feasible for complete resection, therefore, it is associated with better postoperative neurological function and a low chance of recurrence [16].

## Conclusions

We present a case of successful surgical management of multiple spinal PAs contained within a syrinx. Surgeons are advised to aim for a complete resection of tumors with a clear POD. Considerations should be given to tumor histopathology and presenting neurological symptoms as they predict the outcome and recurrence of IMSCT.
